# From Laboratory Screening to a Site-Specific Pilot Field Assessment: Performance of *Brassica oleracea* var. *acephala* ‘Pigeon’ on Mining-Impacted Soils

**DOI:** 10.3390/plants15162482

**Published:** 2026-08-16

**Authors:** Evelyn Terez Polyak, Valer Micle, Ioana Monica Sur

**Affiliations:** Department of Environment Engineering and Entrepreneurship of Sustainable Development, Faculty of Materials and Environmental Engineering, Technical University of Cluj-Napoca, 103–105 Muncii Avenue, 400 641 Cluj–Napoca, Romania; evelyn.polyak@imadd.utcluj.ro

**Keywords:** mining-affected soils, *Brassica oleracea* var. *acephala*, kale, field assessment, root-associated metal retention

## Abstract

Mining-affected soils differ markedly in their physicochemical properties and metal loads, complicating the selection of plant material for phytoremediation. This study evaluated *Brassica oleracea* var. *acephala* ‘Pigeon’ growth across 14 mining-affected soils and a growth reference commercial substrate, followed by a site-specific pilot field assessment on a selected multi-metal-contaminated soil from Cavnic, Romania. Plant establishment, vegetative performance, pooled plant mass, and metal partitioning were assessed under controlled and field conditions. Plant responses varied strongly among soils, and final germination at 30 days after sowing did not reliably predict subsequent vegetative performance. On the selected Cavnic soil, establishment was lower in the field than under controlled conditions, whereas plants that established in situ attained greater final size. Within the seven selected field-grown plants, root-associated concentrations exceeded aboveground concentrations for Cu, Zn, Pb, and Mn. The results show that multi-soil laboratory screening and field assessment capture complementary biological components of plant performance.

## 1. Introduction

Mining activities can leave long-lasting soil contamination with potentially toxic metals, restricting the ecological functioning and future use of affected land [1,2,3]. In mining regions, the environmental legacy is determined not only by total metal concentrations, but also by soil properties that influence root development, water availability, nutrient cycling, and the relative mobility of contaminants [4]. These factors complicate the selection of plant material for phytoremediation or phytomanagement, particularly where soils differ markedly over short spatial distances [4]. In Romania, the closure and remediation of former mining areas remain important environmental challenges, and the establishment of a stable vegetation cover is often a necessary first step toward the functional recovery of disturbed land [2]. Plant-based approaches are therefore of interest because they may support soil stabilization and ecological recovery while avoiding the extensive disturbance associated with conventional remediation methods. However, plant performance depends on the interaction among plant genotype, biomass production, rooting behavior, soil physicochemical properties, and the phytoavailable fraction of metals rather than on total soil concentrations alone [4,5,6].

Controlled experiments are valuable for the preliminary screening of plant species and plant–soil combinations. Nevertheless, laboratory and semi-controlled studies cannot fully reproduce the complexity of field conditions in mining-affected sites, including soil heterogeneity, natural moisture fluctuations, climatic variability, unrestricted root development, and the spatial distribution of contaminants [7]. Field studies have shown that root development, plant biomass, and contaminant uptake may differ substantially between controlled and in situ conditions [7,8,9]. Consequently, laboratory screening should be regarded as a first selection step rather than as a complete predictor of field performance. Site-specific field assessment is needed to determine whether plants that establish under controlled conditions can also survive, develop, and maintain relevant growth under real soil and environmental conditions [10].

Species of the genus *Brassica* have frequently been investigated for phytoremediation because of their relatively rapid growth and high biomass production potential, and agronomic accessibility [11,12,13].

However, review studies indicate that the available evidence is variable and sometimes contradictory. In particular, the field performance of *Brassica* species and the practical feasibility of phytoextraction remain insufficiently documented [12]. For *Brassica oleracea* var. *acephala*, published information is still limited. Recent studies have focused mainly on Cd tolerance and molecular responses under controlled conditions, rather than on whether responses observed in the laboratory can be transferred to multi-metal-contaminated mining soils under field conditions [14]. At the same time, studies performed directly on mining-affected soils show that metal accumulation, translocation, and the practical relevance of a given species are strongly shaped by the edaphic context and must be interpreted under field-relevant conditions [10,15,16]. However, their practical performance is strongly dependent on the metal, soil conditions, cultivar, and experimental setting. Studies on kale and related *Brassica oleracea* materials have reported responses to Cd and Pb exposure under saline, agricultural, or experimentally contaminated soil conditions, showing that metal uptake and tolerance may vary considerably among plant–soil systems [17,18,19]. At the same time, reviews of *Brassica*-based phytoremediation indicate that evidence for efficient phytoextraction under field conditions remains limited, particularly when high biomass production must be combined with substantial translocation of metals to harvestable aboveground tissues [12,13,20]. For *Brassica oleracea* var. *acephala*, recent research has focused mainly on physiological or molecular responses to individual metals under controlled conditions, while comparative evidence across heterogeneous mining soils and subsequent field assessment remains limited [14].

Against this background, the present study assessed *Brassica oleracea* var. *acephala* ‘Pigeon’ across 14 mining-affected soils and a reference commercial substrate, followed by site-specific pilot field assessment on a selected soil from Cavnic, Romania. The objectives were: (i) to compare plant establishment and vegetative performance across contrasting mining-affected soils; (ii) to examine whether early germination predicts final plant development; (iii) to evaluate the extent to which laboratory results are reflected under field conditions; and (iv) to characterize root-associated and aboveground metal concentrations and the corresponding descriptive TF and BCF ratios in selected field-grown plants. This study was intentionally designed as an exploratory, stepwise framework combining controlled multi-soil screening with site-specific pilot field assessment and it was not designed to identify a single causal soil factor, establish diagnostic tissue-concentration ranges, or define normal TF or BCF values for the cultivar. Its main contribution is to show that laboratory screening and field assessment capture complementary biological components of plant performance.

## 2. Results

### 2.1. Laboratory Establishment and Vegetative Performance Across Soils

**Figure 1:**
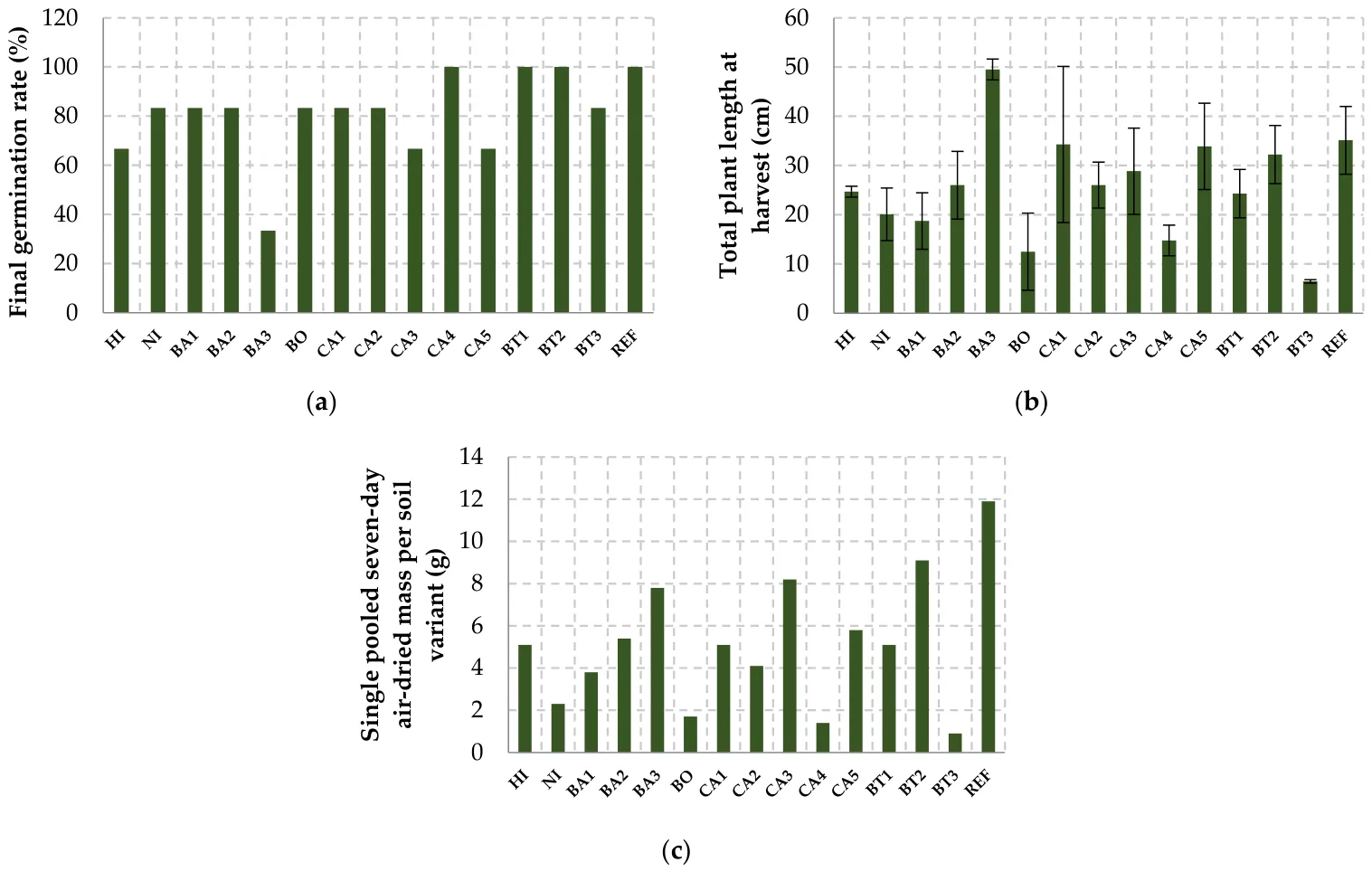
Laboratory response of *Brassica oleracea* var. *acephala* ‘Pigeon’ across mining-affected soil variants and the growth reference commercial substrate: (**a**) final germination rate, expressed as the percentage of germinated seeds from six seeds sown per soil variant; (**b**) total plant length at harvest, presented as the mean ± standard deviation across surviving plants; (**c**) single pooled seven-day air-dried mass value obtained after combining all surviving plants harvested from the three pots of each soil variant. Because material was pooled before weighing, no independent pot-level mass replicates or error estimates were available for panel (**c**).

Plant establishment at 30 days after sowing and subsequent vegetative performance varied markedly among the soil variants. It ranged from 2/6 seeds (33.3%) in BA3 to 6/6 (100%) in CA4, BT1, BT2, and REF. Intermediate germination (4/6 or 5/6) was observed in the remaining mining-affected soils. The observed variation in final germination, total plant length at harvest, and pooled seven-day air-dried mass is illustrated in Figure 1.

Visual monitoring showed that differences in plant development became progressively clearer during the laboratory experiment. BA3, CA3, CA5, BT2, and REF supported comparatively stronger vegetative development, whereas BO, CA4, and BT3 consistently showed restricted growth. The contrast between BA3 and CA4 was particularly pronounced, BA3 had the lowest final germination but produced the tallest plants, whereas CA4 showed complete germination but limited final growth.

**Figure 2:**
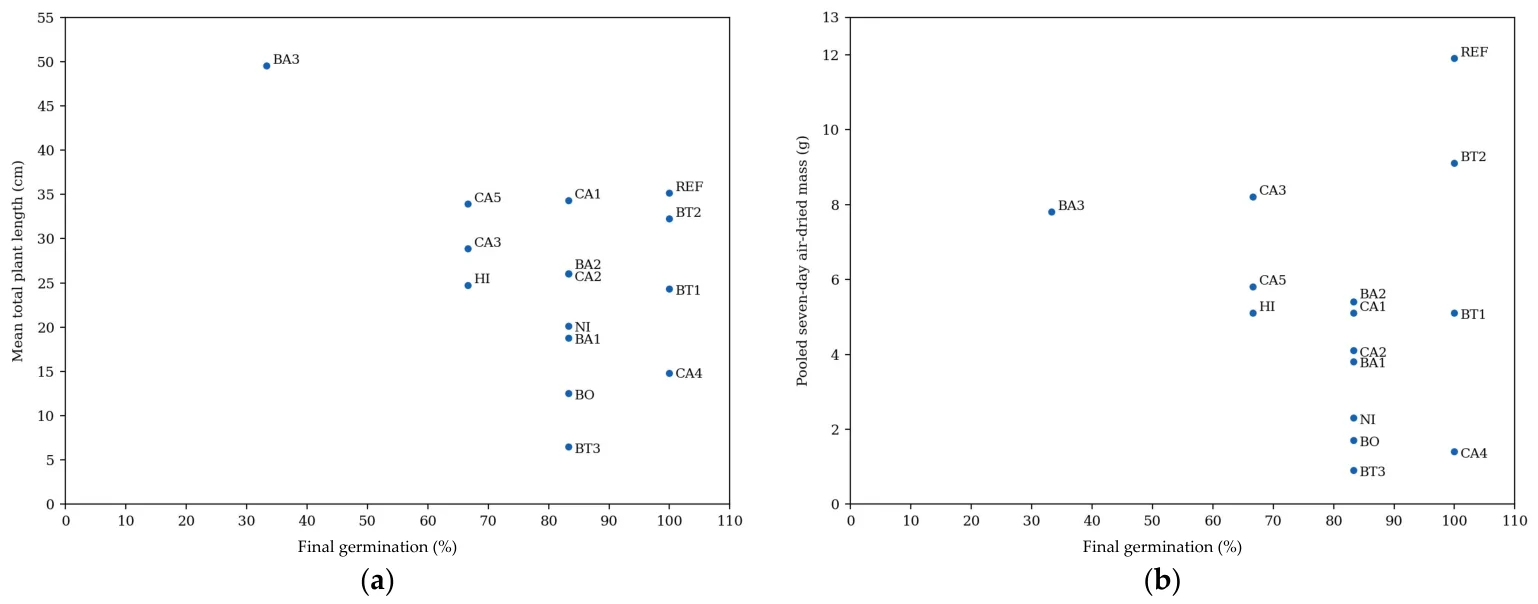
Relationship between final germination and subsequent vegetative performance of *Brassica oleracea* var. *acephala* ‘Pigeon’ across mining-affected soil variants and the growth reference commercial substrate. (**a**) Final germination rate versus mean total plant length at harvest. (**b**) Final germination rate versus pooled seven-day air-dried mass. Each point represents one soil variant. Spearman rank correlations were non-significant for both relationships.

Final biometric measurements confirmed marked variation among soil variants. Mean total plant length ranged from 6.47 ± 0.35 cm in BT3 to 49.50 ± 2.12 cm in BA3 (Figure 2). Among the mining-affected soils, relatively high total plant lengths were also recorded in CA1, CA5, and BT2, whereas BO, CA4, and BT3 showed the lowest values. Root, stem, and leaf measurements followed the same general pattern, with stronger development in BA3, CA1, CA5, and BT2 and reduced growth in BO and BT3.

Pooled seven-day air-dried mass ranged from 0.9 g in BT3 to 11.9 g in REF. Among the mining-affected soils, the highest pooled mass values were recorded in BT2, CA3, BA3, and CA5, whereas BT3, CA4, and BO showed the lowest values. Because each soil variant yielded one composite seven-day air-dried mass value and constant dry mass was not verified, these measurements are presented descriptively and are not compared gravimetrically with field oven-dry biomass.

At the pot level, live seedling counts at 30 days after sowing did not show a global difference among soil variants (Kruskal–Wallis H = 15.25; Monte Carlo permutation *p* = 0.401). For post-establishment total plant length, individual values within each pot were averaged; pot-level means showed a global difference among soil variants (H = 33.50; permutation *p* < 1 × 10^−5^), without pairwise post hoc comparisons. Root, stem, and leaf lengths were retained as descriptive individual-plant measurements. At the soil-variant level, 30 days after sowing establishment was not significantly associated with pooled seven-day air-dried mass (Spearman ρ = −0.11, *p* = 0.688) or mean total plant length (ρ = −0.24, *p* = 0.388). Pooled seven-day air-dried mass was positively associated with mean total plant length (ρ = 0.864, *p* = 3.31 × 10^−5^); this exploratory association should be interpreted cautiously because mass was available as one pooled value per soil and was influenced by the number of harvested plants.

Overall, the laboratory screening showed that germination and final vegetative performance did not follow the same pattern across soil variants. Comparable heterogeneity in *Brassica* performance has been reported under contrasting contaminated-soil conditions and metal exposures [12,13,18].

### 2.2. Observed Relationship Between Soil Properties and Plant Performance

The observed relationships between baseline soil properties and plant response did not indicate a single edaphic variable as the main determinant of performance. Soil pH and electrical conductivity showed no significant monotonic correlations with final germination rate, mean total plant length, or pooled seven-day air-dried mass (Spearman, all *p* > 0.40). These non-significant associations should not be interpreted as evidence that pH or EC were biologically unimportant; the design did not separate their effects from those of other measured and unmeasured soil factors.

Soil texture alone also did not explain the observed variation in plant performance. Most mining-affected soils had a loamy texture but supported markedly different levels of establishment and vegetative development. In addition, the two sandy clay soils showed contrasting responses: CA5 supported relatively strong growth, whereas BT3 produced the weakest overall vegetative response.

When soil variants were grouped according to their Sekera structural classes [21], variants classified as well structured or very well structured generally showed greater mean total plant length and pooled seven-day air-dried mass, whereas poorer structural conditions more often coincided with limited growth. These class-level patterns are presented descriptively because the groups were small and unbalanced, and one structural class contained only one soil variant.

The descriptive pattern suggests that structural condition may have contributed to vegetative performance, but the present design does not establish soil structure as the principal determinant. Soil structure can affect aeration, water movement, root penetration, and the distribution of nutrients and contaminants in the root zone [5,22]. The observed variation therefore cannot be attributed to structure, pH, electrical conductivity, pseudo-total metal concentrations, or any other individual descriptor.

### 2.3. Field Establishment and Vegetative Performance

Seedling emergence was first observed 16 days after sowing. Twelve seedlings emerged from the 30 seeds sown, corresponding to an emergence rate of 40.0%, and all 12 emerged plants survived until the final assessment at 150 days after sowing. Thus, no post-emergence mortality was recorded during the pilot assessment. Field emergence was lower than laboratory germination on CA5 soil, where four of six seeds germinated (66.7%). The two percentages are comparative establishment indicators rather than identical germination endpoints.

Aboveground height was monitored for all 12 surviving plants. Mean height increased from 7.9 cm at 50 days after sowing to 27.1 cm at 100 days after sowing, 30.4 cm at 120 days after sowing, and 34.8 cm at 150 days after sowing. Plant height ranged from 19 to 35 cm at 100 days after sowing, from 20 to 39 cm at 120 days after sowing, and from 23 to 46.5 cm at 150 days after sowing.

Seven of the 12 surviving plants were selected for detailed biometric and chemical assessment using spatially stratified purposive sampling, with one plant obtained from each of seven equal longitudinal subzones of the plot. The selected plants were also chosen to span the observed size range. Consequently, the measurements reported for V1–V7 describe the analyzed subset and should not be interpreted as unbiased estimates of all surviving plants or as independent plot-level replicates. Among the seven analyzed plants, total plant length ranged from 40.0 to 66.0 cm, and oven-dry biomass ranged from 12.18 to 549.45 g. The substantial variation illustrates the heterogeneity within the analyzed subset of the single pilot plot. Because all seven observations originated from one plot and the analytical subset was purposively selected, this variation cannot be attributed quantitatively to bulk soil-metal concentrations or to any other individual soil property. Summary biometric characteristics of the seven field-grown plants harvested from CA5 soil are presented in Table 1.

**Figure 3:**
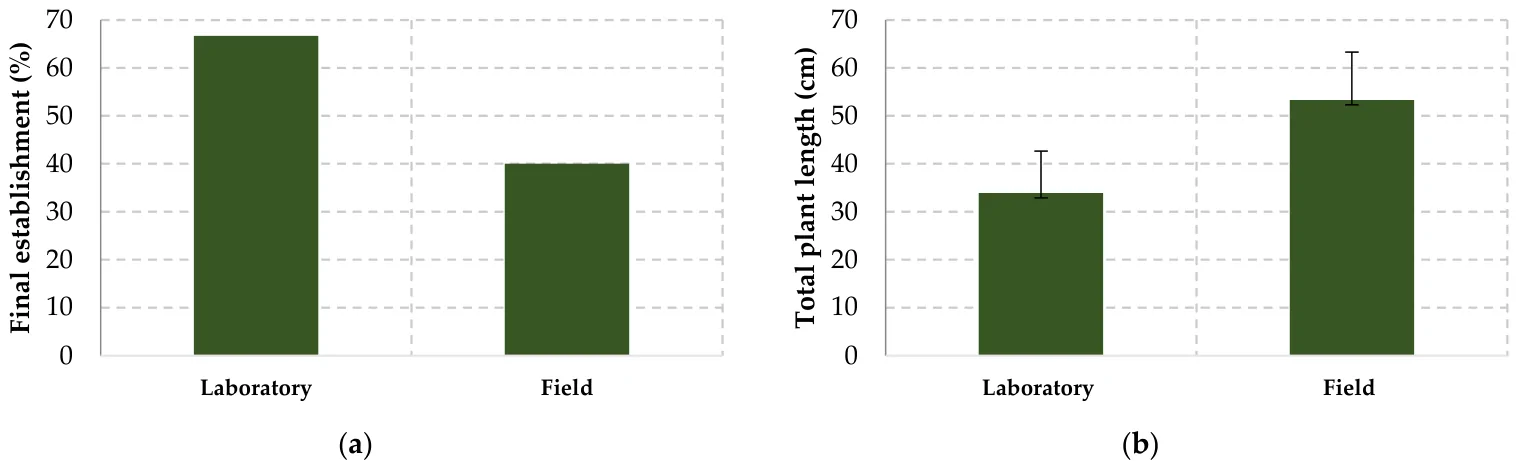
Comparison of laboratory and field performance on CA5 soil. (**a**) Laboratory germination on CA5 soil (4/6 seeds) and field emergence (12/30 seeds); all 12 field-emerged plants survived to harvest. (**b**) Final total plant length of laboratory-grown CA5 plants and field-grown plants V1–V7. Bars representing laboratory and field summary values show the mean ± standard deviation. The field total-length bar describes the seven purposively selected plants V1–V7 from one unreplicated plot and is not an estimate based on independent field replicates.

The broad within-plot variation indicated that plants which successfully established under field conditions differed substantially in their final vegetative development. The comparison between establishment and final plant size under laboratory and field conditions on CA5 soil is shown in Figure 3.

CA5 showed lower establishment in the field than laboratory, whereas the purposively selected field-grown plants attained greater final size than laboratory-grown CA5 plants. This comparison is descriptive because the experimental stages differed in duration, environmental conditions, analytical endpoints, and sampling structure. A comparable divergence between controlled screening and site-level plant performance has been reported in field-oriented phytoremediation studies [7,8,23].

### 2.4. Descriptive Metal Concentration Ratios and Field Compartment Patterns

Laboratory BAC values varied among metals and soil variants (Table A1). BAC values above 1 were observed mainly for Cr, Cu, Zn, and Ni, whereas Co, Cd, Pb, and Mn generally remained below 1. Because BAC was calculated using pseudo-total soil concentrations and whole-plant laboratory material that included washed roots, it is interpreted only as a descriptive concentration ratio. The value 1 was not treated as a cultivar-specific normal or diagnostic threshold, and BAC was not used as evidence of metal removal, phytoavailability, or phytoextraction efficiency.

**Figure 4:**
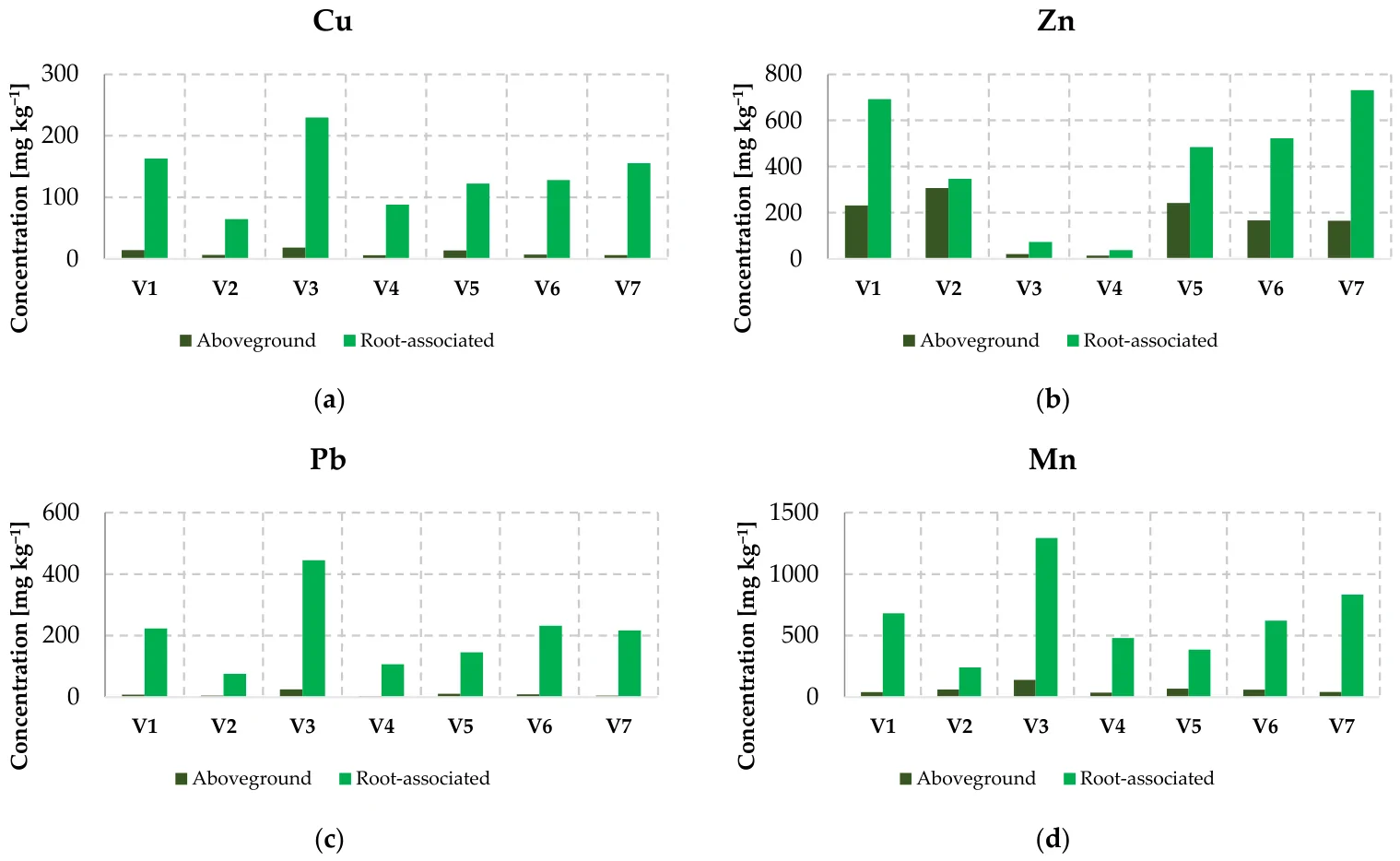
Root-associated and aboveground concentrations of (**a**) Cu, (**b**) Zn, (**c**) Pb, and (**d**) Mn in field-grown *Brassica oleracea* var. *acephala* ‘Pigeon’ plants cultivated on CA5 soil. Note: Root-associated concentrations may include an unknown contribution from fine soil particles remaining after washing. The seven plants are paired within-plot observations from one unreplicated pilot plot.

The root–shoot distribution of Cu, Zn, Pb, and Mn in field-grown plants is illustrated in Figure 4.

Field-grown plants showed a predominance of root-associated metal concentrations. For Cu, Zn, Pb, and Mn, root-associated concentrations exceeded aboveground concentrations in all seven evaluated plants. The exact paired Wilcoxon signed-rank test gave W = 0 and an unadjusted two-sided *p* = 0.0156 for each metal. These results describe a consistent direction among seven paired observations within one unreplicated pilot plot and are not evidence of site-level replication.

Ni was quantified only in roots from two plants and was not detected in aboveground tissues. Cr was quantified in all root samples but only in two aboveground samples, whereas Co was not detected in either plant compartment. Cd was quantified in both compartments in only two plants.

Calculated TF and BCF ratios varied among metals and individual plants (Table S7). TF ranged from 0.013 to 0.883 in quantifiable root–shoot pairs, whereas BCF also varied among plant–metal combinations. These ratios are reported descriptively; no uncontaminated organ-specific control or cultivar-specific normal TF/BCF range was established, and the arithmetic value 1 was not treated as a diagnostic biological threshold.

Within the analyzed subset, aboveground concentrations were lower than root-associated concentrations for Cu, Zn, Pb, and Mn. In comparison with the laboratory stage, where plant tissues were analyzed as composite whole-plant samples, the field stage provided compartment-specific analytical observations. These data do not establish a mechanism of internal accumulation, a normal root-to-shoot relationship, or a defined phytoremediation function. Root-associated and aboveground metal concentrations and descriptive TF and BCF ratios derived in field-grown *Brassica oleracea* var. *acephala* ‘Pigeon’ plants from CA5 soil are presented in Table A2.

## 3. Discussion

### 3.1. Establishment and Final Vegetative Performance

The laboratory screening showed marked variation in the response of *Brassica oleracea* var. *acephala* ‘Pigeon’ among the investigated soil variant. Differences among soils were evident not only in germination, but also in final plant size and pooled seven-day air-dried mass. The data demonstrate contrasting plant–soil combinations, but they do not identify which measured or unmeasured soil factor caused the observed responses. Pseudo-total metal concentrations and individual soil descriptors should therefore be interpreted as contextual variables rather than causal predictors.

A descriptive tendency toward stronger vegetative development in well-structured and very well-structured variants was observed, but the structural groups were too small and unbalanced for a robust class-level test. Soil structure can influence aeration, water retention and movement, root penetration, and the spatial distribution of nutrients and contaminants in the root zone [5,22]. Therefore, the absence of clear relationships between plant performance and pH or electrical conductivity considered separately does not indicate that these variables were unimportant; rather, it suggests that they acted within a broader edaphic context.

The investigated soils represented naturally heterogeneous multi-element systems rather than controlled single-factor treatments. Potential synergistic or antagonistic interactions among metals, together with pH-dependent changes in availability and un-measured differences in nutrients, organic matter, and exchange properties, could not be separated experimentally. The absence of a single significant monotonic relationship should therefore not be interpreted as evidence that individual elements or pH were biologically unimportant; rather, the design does not support causal attribution among these overlapping factors.

A central result of the laboratory stage was the partial separation between early establishment and final vegetative performance. BA3 showed the lowest germination rate but produced the tallest plants, whereas CA4 showed complete germination but limited subsequent growth. The date-specific establishment count therefore acted as an initial screening indicator but did not reliably predict final vegetative performance. This distinction is important for *Brassica*-based phytoremediation assessments, in which tolerance, biomass production, and the capacity to maintain growth over time must all be considered together [12,18,20].

The BA3 response may reflect selective establishment, reduced competition among the surviving plants, or other unmeasured edaphic factors. These interpretations remain hypotheses because seed-level vigor, nutrient availability, and extractable metal fractions were not determined.

From a practical perspective, these findings indicate that preliminary screening should evaluate at least two distinct phases: establishment capacity and sustained vegetative development. A cultivar may germinate successfully but remain poorly productive under a given soil condition, as observed for CA4. Conversely, limited establishment may be followed by strong growth of the plants that survive, as observed for BA3. For *B. oleracea* var. *acephala* ‘Pigeon’, the multi-soil approach was therefore valuable because it identified contrasting plant–soil responses that would not have been apparent from germination data alone.

### 3.2. Laboratory Screening and Field Assessment Captured Complementary Biological Phases

The comparison between the laboratory and field stages on CA5 soil showed that the two experimental systems captured different components of plant performance. The laboratory stage provided a controlled comparison of establishment and vegetative response across contrasting soils, whereas the field stage assessed establishment under site conditions and the subsequent development of plants that successfully survived. On CA5, final establishment was lower in the field than under laboratory conditions, while the purposively selected field-grown plants attained greater observed final size. Because the field subset was non-random and the stages differed in design, this size comparison is descriptive only.

These outcomes should not be interpreted as contradictory. Instead, they indicate that early establishment and subsequent vegetative development were influenced by partly different environmental constraints. Under laboratory conditions, plant response was evaluated within a limited and standardized rooting volume, with regular watering and reduced environmental variability. In the field, seedling establishment occurred under greater spatial and temporal variation in soil surface conditions, moisture dynamics, and microenvironmental conditions. However, plants that successfully established in the field had access to a larger rooting volume and to soil–plant interactions that could not be reproduced fully under pot conditions. Comparable differences between controlled experiments and field-scale plant performance have been reported in phytoremediation studies, particularly where root development, soil heterogeneity, and local environmental conditions influence plant growth and contaminant behavior [7,8,23].

The field stage was intentionally designed as a site-specific pilot assessment rather than as a direct replication of the laboratory experiment. Its purpose was to document whether CA5 could support the establishment and subsequent growth of plants under field conditions. In this respect, the laboratory stage acted as an initial screening step, whereas the field stage provided ecological assessment of the post-establishment response.

The lower field emergence cannot be attributed exclusively to the properties of CA5 soil. Seeds were surface-sown, lightly pressed into the soil, left uncovered, and not artificially protected. In addition, abundant rainfall occurred during the early establishment period. Rainfall-related seed displacement, localized burial, soil-surface crusting, transient water saturation, predation, and microsite heterogeneity may therefore have contributed to the lower emergence recorded under field conditions. These mechanisms were not monitored separately and cannot be distinguished by the present design. Because all 12 seedlings that emerged survived to harvest, the difference between laboratory and field performance occurred primarily during the pre-emergence and emergence stage rather than through subsequent seedling mortality.

The comparison between the two systems should be based primarily on establishment and final plant size rather than on direct biomass equivalence. Laboratory material was processed as pooled screening material and air-dried for seven days, whereas the larger field plants were oven-dried at 60 °C for 72 h for compartment-specific assessment. These drying procedures were not standardized, constant mass was not verified for laboratory material, and residual moisture—particularly in roots—cannot be excluded. Laboratory mass is therefore reported only as pooled seven-day air-dried mass. In addition, limited organ-specific mass in the laboratory would have produced aliquots substantially below the 3 g target and higher sample-specific reporting limits, so roots, stems, and leaves were combined. Field plants provided sufficient material for separate root-associated and aboveground analyses. The two stages consequently address different analytical endpoints, and neither gravimetric mass nor internal metal partitioning is compared quantitatively between them.

These datasets therefore provide complementary information on vegetative performance but are not strictly equivalent gravimetric measurements. Nevertheless, the greater final size of field-grown surviving plants demonstrates that lower establishment did not prevent substantial vegetative development under site conditions.

This distinction has practical implications for phytoremediation-oriented plant selection. Candidate plants should not be evaluated only by germination success or by performance in controlled pot experiments. Their suitability depends both on their capacity to establish under the initial constraints of contaminated soil and on their ability to maintain growth after establishment under field conditions. Stepwise designs that combine controlled screening with field assessment can therefore provide a more realistic basis for interpreting plant performance on heterogeneous mining-affected soils [8,9,10].

### 3.3. Interpretation of Root-Associated and Aboveground Metal Concentrations

The laboratory BAC values varied among soil variants and elements. Values above 1 occurred mainly for Cr, Cu, Zn, and Ni, whereas Co, Cd, Pb, and Mn generally remained below 1. However, the value 1 is an arithmetic ratio and was not established experimentally as a normal or diagnostic threshold for this cultivar. The soil concentrations used in these calculations were obtained after aqua regia digestion and therefore represented pseudo-total rather than directly phytoavailable metal fractions. In mining-affected soils, the relationship between analytically determined soil concentrations and plant uptake may be influenced by soil properties, metal speciation, and rhizosphere processes [5,24,25]. BA2 and BA3 illustrate the limits of interpretation from a single descriptor: BA3 had substantially higher EC than BA2, but the contrasting responses cannot be assigned to salinity, pseudo-total metal concentrations, or pH individually because extractable metals, nutrient status, and other chemical properties were not measured.

BAC should not be interpreted as independent evidence of plant-mediated metal removal. Instead, it provides a descriptive comparison of concentrations measured in whole-plant material and baseline pseudo-total soil, and it identifies patterns requiring validation through bioavailable soil fractions, organ-specific analyses, and replicated field observations. No cultivar-specific normal BAC range was established.

The present experiment did not include an uncontaminated mineral-soil control with organ-specific metal analysis. Consequently, cultivar-specific background root and shoot concentrations and normal TF or BCF ranges could not be established. REF supported comparison of plant establishment and growth only and should not be interpreted as a geochemical or tissue-concentration control.

The field stage distinguished concentrations measured in root-associated samples from those in aboveground tissues. For Cu, Zn, Pb, and Mn, root-associated concentrations were higher in all paired observations. Ni was not detected in aboveground tissues, and Cr was quantified in only two aboveground samples. TF and BCF were retained as within-study descriptive ratios and were not interpreted against cultivar-specific normal ranges.

The near-neutral pH of CA5 (pH 7.31) may have influenced the availability of several metals. However, rhizosphere pH, plant Fe status, and Strategy I root responses were not measured. Consequently, the possible contribution of root-induced acidification to Mn or Zn acquisition cannot be evaluated from the present data.

The measured tissue concentrations cannot be classified experimentally as normal, optimal, sufficient, deficient, or toxic for this cultivar. The study did not include an uncontaminated mineral-soil control with organ-specific tissue analysis, and cultivar-, organ-, age-, and condition-specific diagnostic ranges were not established. In addition, the two experimental stages used different tissue fractions and mass bases. The reported concentrations should therefore be interpreted only as analytical observations within the investigated plant–soil systems rather than as diagnostic thresholds of nutritional sufficiency or metal toxicity.

A strong phytoextraction profile requires not only plant tolerance and biomass production, but also effective transfer of contaminants to harvestable aboveground tissues [12,26]. In the present subset, aboveground concentrations were lower than root-associated concentrations for the four metals with complete paired data. However, this pattern alone does not establish efficient exclusion, metal tolerance, phytoextraction capacity, or phytostabilization. Interpretation is constrained by the absence of an uncontaminated organ-specific control, the lack of diagnostic tissue ranges, and the possibility of residual fine soil particles in root-associated samples.

The higher concentrations measured in root-associated samples do not demonstrate that all metals were internally accumulated, because metals adsorbed to root surfaces and fine residual soil particles may have contributed despite washing. The observed pattern should therefore be described as a compartment-specific analytical observation rather than unequivocal intracellular sequestration. The study also did not measure reduced metal mobility, bioavailability, erosion, or exposure risk over time and consequently does not demonstrate phytostabilization effectiveness [10,27,28].

The present data do not support classification of *Brassica oleracea* var. *acephala* ‘Pigeon’ as either an efficient phytoextractor or a demonstrated phytostabilizer under the investigated conditions. Replicated field trials that include an uncontaminated mineral-soil control, organ-specific background concentrations, bioavailable metal fractions, and physiological indicators would be required to define cultivar-specific responses and phytoremediation relevance.

### 3.4. Scope of the Study and Implications for Future Work

The present study was intentionally designed as a stepwise framework combining controlled multi-soil screening with a site-specific pilot field assessment. Its purpose was not to demonstrate remediation efficiency or identify a single causal edaphic driver, but to document variation in establishment, vegetative development, and compartment-specific metal concentrations across the investigated plant–soil combinations. Accordingly, the findings should be interpreted as exploratory observations rather than direct estimates of site-wide remediation performance or diagnostic cultivar responses.

Several aspects define the scope of this interpretation. The laboratory stage involved distinct soil samples rather than replicated site-level sampling, and uneven plant survival among variants limited the strength of confirmatory inference. In the field stage, the seven analyzed plants represented within-plot observations from one selected site and should not be interpreted as independent plot-level replicates. Laboratory plant mass was measured as pooled seven-day air-dried mass, whereas field biomass was oven-dried; the datasets therefore do not support a gravimetrically equivalent comparison. The laboratory analysis of pooled whole-plant material and the separate root-associated/aboveground analysis in field plants likewise preclude a direct quantitative comparison of internal metal partitioning between stages.

The geochemical interpretation was further limited by the use of aqua regia-derived pseudo-total concentrations without complementary extractable or bioavailable fractions. Future work should therefore combine plant response data with measurements of operationally defined metal availability, soil organic matter, cation-exchange capacity, and post-harvest changes in soil properties. Such measurements would allow plant uptake and root-associated retention to be interpreted more directly in relation to soil processes and rehabilitation potential [15,25]. Replicated field plots across contrasting mining soils, standardized biomass-drying procedures, and organ-specific analyses in both laboratory and field stages would further strengthen the comparison between controlled and site conditions.

Future replicated work should test whether establishment, vegetative development, and compartment-specific metal concentrations change under defined soil amendments and controlled reference conditions. Organic or mineral amendments may modify soil physical conditions, nutrient status, and metal mobility while supporting vegetation establishment; however, their effects must be assessed with replicated plots, an uncontaminated mineral-soil control, organ-specific tissue analysis, and measurements of plant physiological status [8,29,30]. The main contribution of the present study is therefore the exploratory documentation of contrasting responses across mining-affected soils and the identification of methodological priorities for subsequent field validation, while showing that laboratory screening and field assessment capture complementary biological phases of plant response, providing a more informative basis for selecting plant material for mining-affected soils than laboratory screening alone.

## 4. Materials and Methods

### 4.1. Study Design and Analytical Logic

The study used a two-stage design combining controlled multi-soil screening with site-specific pilot field assessment. In the laboratory stage, the establishment and vegetative performance of *Brassica oleracea* var. *acephala* ‘Pigeon’ were evaluated across 14 mining-affected soils and one growth reference commercial substrate. The field stage was conducted on one selected soil from Cavnic, Romania, to assess plant establishment, vegetative development, and root–shoot metal partitioning under site conditions.

The laboratory stage was used to identify relative differences among plant–soil combinations during establishment and vegetative development. The field stage was used to evaluate whether plants that successfully established on the selected soil could maintain growth under real site conditions. Soil metal concentrations determined after aqua regia digestion were treated as pseudo-total concentrations and were used for comparative interpretation rather than as direct measures of phytoavailable metal fractions [5,25].

The bioaccumulation coefficient (BAC), the translocation factor (TF), and the bioconcentration factor (BCF) were retained as within-study descriptive concentration ratios. They were not treated as direct estimates of remediation efficiency, diagnostic indicators of toxicity or sufficiency, or stand-alone criteria for classifying the cultivar. No cultivar-specific normal ranges were established for these ratios.

### 4.2. Plant Material

The ornamental kale cultivar *Brassica oleracea* var. *acephala* ‘Pigeon’ was used in both the laboratory and field stages. Seeds were purchased from a commercial seed supplier in Romania (Agro Group BN SRL, Unirea Bistrița, Bistrița-Năsăud, Romania). The same seed lot was used in both experimental stages.

### 4.3. Soil Samples and Baseline Characterization

Surface soil samples were collected from the 0–10 cm layer in areas affected by historical mining activities. This depth was selected because the surface soil layer is most directly involved in seed germination, early seedling establishment, and the initial root–soil interaction, while also representing the zone commonly exposed to anthropogenic metal inputs [5,31]. The samples were coded according to their sampling area: HI (Handalul Ilbei), NI (Nistru), BA1–BA3 (Baita), BO (Bozanta Mare), CA1–CA5 (Cavnic), and BT1–BT3 (Baiut).

A commercial horticultural substrate, COMPO SANA^®^ Universal Potting Soil (COMPO GmbH, Münster, Germany), was used as the growth reference commercial substrate (REF) [32]. According to the product label, the substrate contained peat-based growing material, perlite, limestone, incorporated fertilizer, and a root-growth activator. The manufacturer-declared pH range was 5.0–6.5, measured in CaCl_2_, and the declared salinity was below 3.0 g L^−1^ [32]. These product specifications were not independently verified using the analytical procedures applied to the mining-affected soils. REF was included only as a biological growth benchmark. It was not a mineral-soil control and was not used to establish background tissue concentrations, normal root-to-shoot relationships, or reference TF and BCF ranges for the cultivar.

Before the laboratory experiment, soils were air-dried and homogenized. Soil texture, soil structure, pH, electrical conductivity, and pseudo-total concentrations of Ni, Cr, Co, Cu, Zn, Cd, Pb, and Mn were determined before sowing. These properties were selected because they may simultaneously influence plant development and metal behavior in soil, including metal mobility and relative availability [30,33]. Soil pH and electrical conductivity were determined in a 1:10 (w/v) soil-to-distilled-water suspension after a 2 h equilibration period. Soil pH was measured using a calibrated glass-electrode pH meter (Hanna Instruments, Nusfalău, România), and EC was determined using a conductivity meter (Hanna Instruments, Nusfalău, România). Coarse particle-size distribution was assessed by dry sieving (Retsch GmbH, Haan, Germany), whereas soil structural condition was evaluated using the Sekera water-dispersion method. Baseline texture, soil structural condition, pH, and electrical conductivity of the investigated soil variants are presented in Table 2. The complete multi-depth soil-contamination dataset was subsequently published separately [34]. Table S1 reports only the baseline topsoil values corresponding to the soil materials used in the present plant experiment. These previously characterized soil data are included here for experimental context and are not presented as a new contamination survey [34]. Easily extractable metal fractions, organic matter, CEC, available phosphorus, and rhizosphere pH were not determined.

Pseudo-total soil metal concentrations were determined after aqua regia digestion. These values were used as comparative geochemical descriptors and were not interpreted as direct measures of metal phytoavailability [15,33].

Each soil variant represented a distinct soil sample and was not treated as a replicate of its sampling area. Accordingly, comparisons focused on relative plant responses across plant–soil combinations rather than on estimating the full spatial variability of the mining sites.

### 4.4. Laboratory Screening Experiment

The laboratory experiment was conducted in recyclable plastic pots with a diameter of 10 cm and a height of 7.5 cm. Three pots were prepared for each soil variant and for the REF growth reference substrate, with 300 g of material placed in each pot.

Two seeds of *B. oleracea* var. *acephala* ‘Pigeon’ were sown in each pot, resulting in six seeds per variant. The seeds were placed on the soil surface and were not covered, in order to allow light access during germination.

Before sowing, each pot was watered with approximately 50 mL of tap water. Pots were maintained on a north-facing laboratory windowsill and received natural light for approximately 14.5 h per day. Pots were watered every three days using equal volumes of tap water, and their positions were periodically changed to reduce local microenvironmental effects.

The laboratory stage lasted 140 days. The pot was considered the cultivation unit, whereas individual seeds and surviving plants were used to describe establishment and final plant performance.

### 4.5. Monitoring of Germination and Plant Development

The timing of first seedling emergence was recorded for each soil variant. Germination was assessed at 10 and 30 days after sowing and was expressed as the number of germinated seeds relative to the six seeds sown per variant.

**Figure 5:**
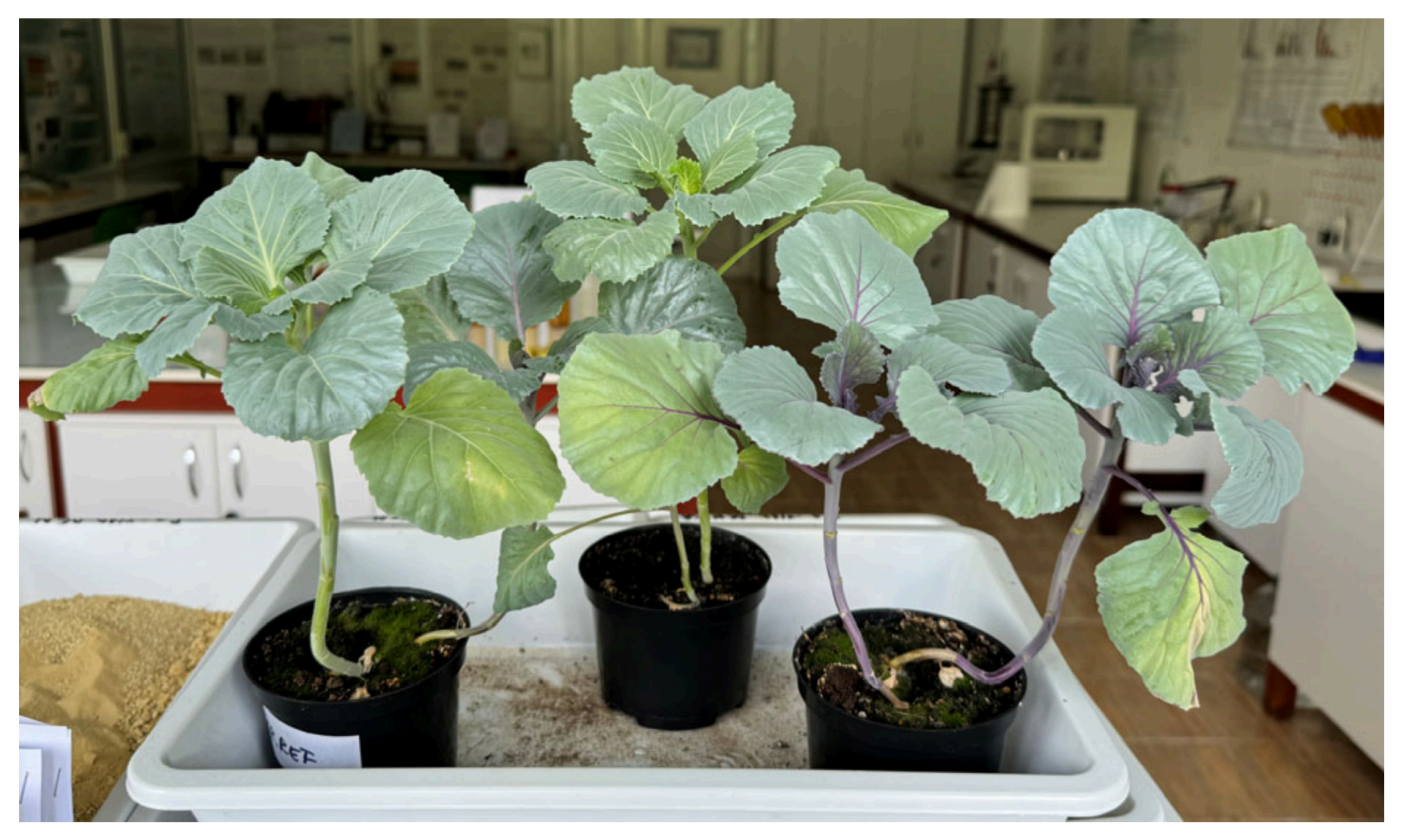
Laboratory experimental setup for the multi-soil screening of *Brassica oleracea* var. *acephala* ‘Pigeon’.

Plant development was monitored at 10, 30, 50, 80, 110, and 140 days after sowing. At each observation time, plant vigor, leaf development, general appearance, and visible stress symptoms were documented. Representative laboratory growing conditions are shown in Figure 5.

After 80 days, dried basal leaves were removed, and subsequent observations were made after this step.

### 4.6. Biometric Measurements and Seven-Day Air-Dried Mass

At the end of the laboratory experiment, surviving plants were harvested and cleaned of adhering soil particles. Plant materials were washed with tap water and then with distilled water.

Root length, stem length, leaf length, and total plant length were measured. After washing, laboratory-grown plant material was air-dried for seven days at approximately 21 °C and 40% relative humidity under the same conditions for all soil variants. Constant dry mass was not verified and residual moisture cannot be excluded. The resulting measurement is therefore reported as pooled seven-day air-dried mass rather than oven-dry biomass. No quantitative gravimetric comparison was made with field material, which was oven-dried at 60 °C for 72 h. For each soil variant, all harvested plants from the three pots were combined to obtain one pooled seven-day air-dried mass value.

### 4.7. Determination of Soil and Plant Metal Concentrations

The concentrations of Ni, Cr, Co, Cu, Zn, Cd, Pb, and Mn were determined in soil and plant material. Soil samples collected before the laboratory experiment and at the end of the laboratory stage were analyzed after aqua regia digestion (3:1, *v*/*v*, HCl: HNO_3_; 21 mL: 7 mL). Laboratory plant concentrations are reported on a seven-day air-dried mass basis.

Because laboratory-grown plants produced limited organ-specific biomass, separate digestion of roots, stems, and leaves would have resulted in multiple aliquots substantially below the target analytical mass and higher sample-specific reporting limits. The organs were therefore combined as a whole-plant sample for the laboratory screening. No direct quantitative comparison of internal metal partitioning was made between the laboratory and field stages. Plant material was ground and homogenized before digestion. A target aliquot of 3 g of plant material was placed in a 100 mL Berzelius beaker. When less than 3 g was available, the entire available mass was used. Samples were digested using 21 mL concentrated hydrochloric acid and 7 mL concentrated nitric acid. The beakers were covered with watch glasses and heated on a sand bath (FALC Instruments, Trezzano sul Naviglio, Milan, Italy) until digestion was complete. After cooling, the digests were filtered into 100 mL volumetric flasks and brought to volume with distilled water.

Metal concentrations were determined by inductively coupled plasma optical emission spectrometry using a PerkinElmer Optima 5300 DV ICP-OES system (PerkinElmer, Shelton, CT, USA). Analytical quality control included multi-element calibration standards, reagent and procedural blanks, and the certified reference material ERM-CC018. The accredited laboratory reported recoveries ranging from 97% to 104% for the certified elements in ERM-CC018. ERM-CC018 is a soil certified reference material and does not provide a certified value for Mn or matrix-matched validation for plant tissues.

The instrumental LODs were 0.03 mg L^−1^ for Ni, 0.02 mg L^−1^ for Cr, 0.01 mg L^−1^ for Co, Cu, and Cd, 0.03 mg L^−1^ for Zn and Mn, and 0.04 mg L^−1^ for Pb. For a 3.000 g aliquot diluted to 100 mL, these corresponded to approximate sample-equivalent reporting limits of 1.00 mg kg^−1^ for Ni, 0.67 mg kg^−1^ for Cr, 0.33 mg kg^−1^ for Co and Cu, 1.00 mg kg^−1^ for Zn, 0.33 mg kg^−1^ for Cd, 1.33 mg kg^−1^ for Pb, and 1.00 mg kg^−1^ for Mn. For plant samples with an aliquot below 3 g, the corresponding sample-specific reporting limit increased proportionally as aliquot mass decreased. Instrumental determinations were performed in triplicate, and reported concentrations represent the mean of three analytical readings; these analytical readings were not treated as independent biological replicates.

### 4.8. Indicators Used for the Laboratory Experiment

The bioaccumulation coefficient (BAC) was calculated as follows [10,15,24]:

(1) $$BAC=\frac{C_{plant}}{C_{soil}}$$

where C_plant_ is the metal concentration in plant biomass, and C_soil_ is the metal concentration in soil before the experiment. BAC was retained as a descriptive whole-plant-to-pseudo-total-soil concentration ratio. The arithmetic value 1 was not treated as a cultivar-specific normal or diagnostic threshold, and BAC was not used to infer metal removal or phytoextraction efficiency.

### 4.9. Site-Specific Pilot Field Assessment

The field stage was conducted on CA5 soil from Cavnic, Romania. CA5 was selected purposively for the pilot field assessment because it combined multi-metal contamination (Cr 25.4 mg kg^−1^, Cu 710 mg kg^−1^, Zn 1584.67 mg kg^−1^ and Pb 1192 mg kg^−1^), laboratory germination of 4/6 seeds, comparatively strong post-establishment growth, and practical access to the corresponding field location. This selection was not based on a statistical ranking and the plot was not intended to represent the entire Cavnic mining-affected area.

The experimental plot measured 0.8 m × 4.0 m, corresponding to a total area of 3.2 m^2^. The plot was cleared of existing vegetation and plant residues, after which the soil was loosened and leveled before sowing.

**Figure 6:**
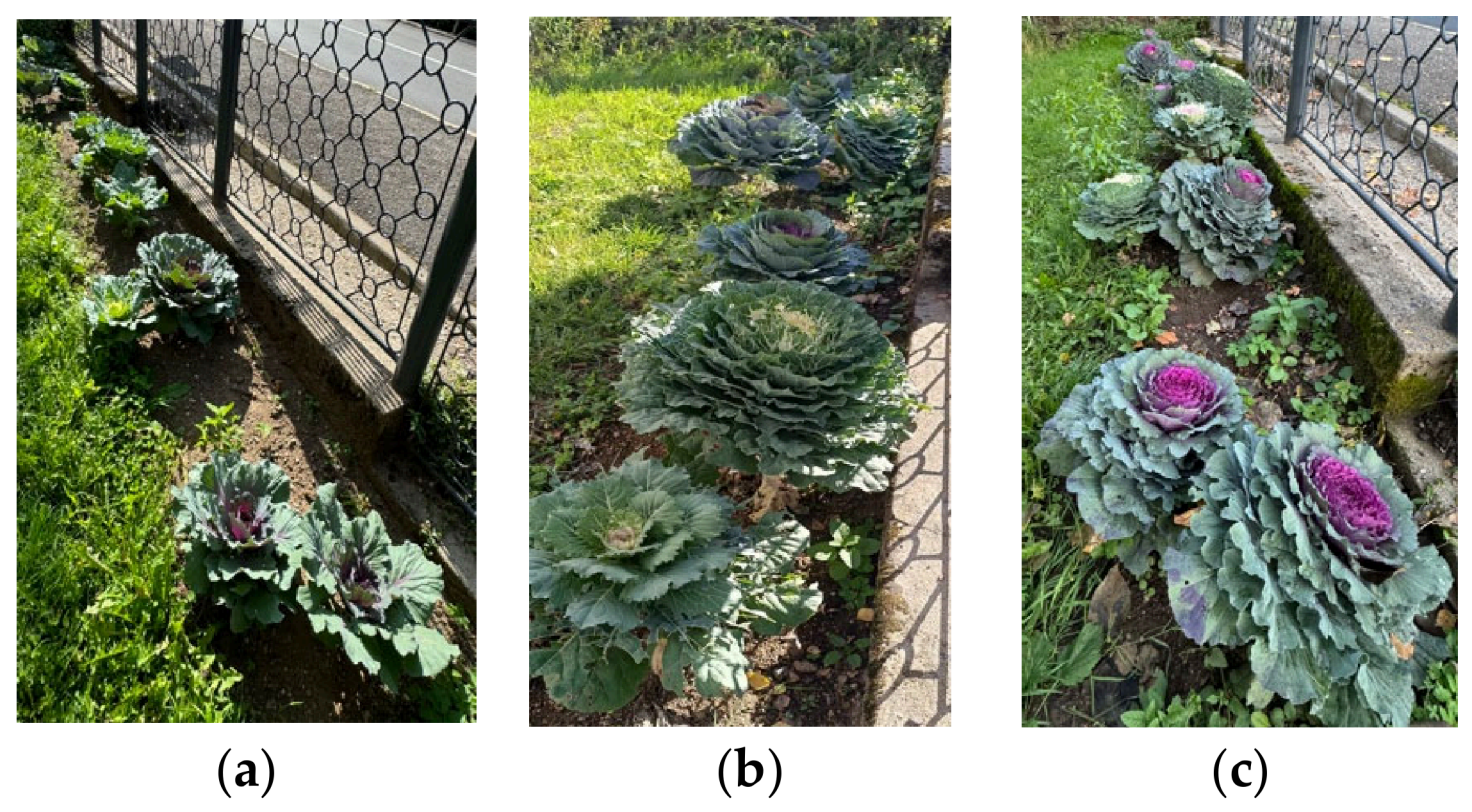
Field site used for the assessment of *Brassica oleracea* var. *acephala* ‘Pigeon’ on CA5 soil in Cavnic, Romania. (**a**) After 100 days from sowing; (**b**) after 120 days from sowing; (**c**) after 150 days from sowing.

Sowing was performed in May 2024 using the same cultivar as in the laboratory stage. A total of 30 seeds were manually sown on the soil surface in two rows, with an approximate spacing of 25 cm within-row spacing and between-row spacing. The seeds were placed at the soil surface, lightly pressed into the soil, and left uncovered. No artificial physical protection was used after sowing. The plot was watered immediately after sowing. During dry periods, plants were watered twice per week using approximately 100 mL of water per plant. Weeds were removed manually throughout the experiment. Seedling emergence was recorded at 16 days after sowing. Aboveground plant height was subsequently measured at 50, 100, 120, and 150 days after sowing for all 12 plants that had emerged. The same plants were followed longitudinally throughout the pilot assessment. The CA5 site-specific pilot field assessment site is shown in Figure 6.

Harvesting was performed in October 2024. Field observations indicated repeated abundant rainfall during the early emergence period. At harvest, the length of the single CA5 pilot plot was divided into seven equal longitudinal subzones to provide spatial coverage of the plot. Five subzones contained two surviving plants, whereas two subzones contained one surviving plant. One plant was selected from each subzone for detailed biometric and chemical assessment. In subzones containing two plants, selection was purposive and was made so that the final analytical subset also spanned the observed range of plant sizes across the plot. The resulting seven plants, coded V1–V7, therefore constituted a spatially stratified subset. They were treated as individual within-plot observations from one unreplicated pilot plot and not as independent field replicates or as an unbiased sample of all 12 surviving plants.

### 4.10. Field Plant Measurements and Compartment-Specific Metal Analysis

Harvesting was performed manually while preserving roots and aboveground parts intact. After harvesting, each plant was cleaned of soil and labeled. Roots were manually freed of coarse soil, washed under tap water, and subsequently rinsed with distilled water. Ti, V, Si, Al, or another soil-particle tracer was not determined; therefore, residual fine soil particles associated with root surfaces cannot be excluded.

Total plant length, root length, stem plus leaf length, mean leaf length, and rosette diameter were measured. Plant tissues were oven-dried for 72 h at 60 °C using a Binder oven (Binder GmbH, Germany), after which oven-dry biomass was determined gravimetrically using a KERN ABJ 220-4M analytical balance (KERN & SOHN GmbH, Balingen, Germany). Roots and aboveground parts were ground separately and analyzed according to the plant digestion and ICP-OES procedures described above.

For field samples, the translocation factor (TF) and bioconcentration factor (BCF) were calculated as descriptive ratios of measured compartment and soil concentration [10,15]. Because the study did not include an uncontaminated mineral-soil control with organ-specific analysis, these ratios were not interpreted as cultivar-specific normal values or diagnostic evidence of toxicity, sufficiency, phytoextraction, or phytostabilization.

The translocation factor (TF) was calculated as follows [15,24,27]:

(2) $$TF=\frac{C_{abovegroundparts}}{C_{roots}}$$

where C_aboveground_ parts represents the metal concentration in leaves and stems, and C_roots_ represents the metal concentration in roots.

The bioconcentration factor (BCF) was calculated as follows [15,24,27]:

(3) $$BCF=\frac{C_{roots}}{C_{soil}}$$

where C_roots_ represents the metal concentration in roots, and C_soil_ represents the metal concentration in soil.

TF and BCF were calculated only for sample–metal combinations for which the concentrations used in the formulas were quantifiable. Values below the applicable sample-specific reporting limit were designated ND and were neither set to zero nor numerically imputed. Any derived index requiring an ND value was reported as NC (not calculated). In addition, high concentrations in the root-associated samples were treated as compartment-specific observations without assuming a single mechanism of internal accumulation. No normal or background TF/BCF range was established for the cultivar.

### 4.11. Statistical Analysis

The statistical analysis was adapted separately for the laboratory experiment and the pilot field assessment, considering data structure, replication level, and the exploratory nature of the study. Data processing, index calculations, and statistical analyses were performed using Microsoft Excel and Python 3.11 with the pandas, NumPy, and SciPy libraries.

In the laboratory screening, the pot was the experimental unit. In the laboratory experiment, germination rate was calculated for each soil variant based on the six seeds sown and was reported both as a percentage and as the number of germinated seeds relative to the total number of seeds sown. Germination was recorded separately for each pot during the monitoring period. For post-establishment total plant length, measurements from plants growing in the same pot were averaged before between-soil analysis; pots without surviving plants were treated as missing for this conditional growth outcome rather than assigned a zero length. Global differences among soil variants in pot-level 30 days after sowing establishment counts and pot-mean total length were evaluated using the Kruskal–Wallis statistic [35]. Significance was estimated using a Monte Carlo permutation procedure based on 500,000 random permutations while preserving the original group sizes [36,37]. Monte Carlo *p*-values were calculated as

(4) $$\frac{\left(r+1\right)}{\left(B+1\right)},$$

where r represents the number of permuted statistics equal to or greater than the observed statistic and B is the number of random permutations [37,38]. A fixed random seed was used to ensure computational reproducibility. No pairwise post hoc tests were performed. Root, stem, and leaf lengths and pooled seven-day air-dried mass were summarized descriptively; individual plants within a pot and instrumental replicates were not treated as independent biological replicates.

Relationships between baseline soil physicochemical properties and plant response were evaluated at the soil-variant level using Spearman’s rank correlation coefficient [39]. REF was excluded from these analyses because it was used only as a commercial horticultural growth benchmark and not as a mineral-soil control.

Soil structural classes [21] were used for descriptive grouping only. No formal class-level hypothesis test was applied because group sizes were small and unbalanced and one class contained a single soil variant.

For field-grown plants, biometric traits and oven-dry biomass were summarized using the mean ± standard deviation, median, minimum, and maximum values. The seven plants were treated as individual observations within the same field plot.

Differences between root-associated and aboveground metal concentrations were evaluated using the exact paired-sample Wilcoxon signed-rank test for metals with quantifiable paired values for all seven plants [40,41]. Exact two-sided *p*-values were reported as unadjusted, exploratory within-plot evidence and interpreted together with the direction and magnitude of all paired differences; they were not treated as confirmatory site-level inference.

Values below the applicable sample-specific reporting limit were designated ND and were neither set to zero nor numerically imputed. Any derived index requiring an ND value was reported as NC (not calculated). BAC, TF, and BCF were calculated only when all values required by the corresponding equation were quantifiable. A significance threshold of *p* < 0.05 was used for descriptive statistical interpretation.

## 5. Conclusions

*Brassica oleracea* var. *acephala* ‘Pigeon’ establishment and subsequent vegetative performance varied markedly among the investigated soil variants and represented partially distinct phases of response. Under laboratory conditions, germination and final plant size or pooled seven-day air-dried mass did not consistently follow the same pattern, indicating that early establishment alone was not sufficient to predict later plant performance across the investigated soils. The experimental design did not identify which individual soil property, metal concentration, or multi-element interaction caused these differences.

The comparison between laboratory screening and the pilot field assessment indicated that the two stages captured complementary observations. Field emergence on CA5 was lower than laboratory establishment, whereas the purposively selected field-grown plants attained greater observed final size. This comparison is descriptive because the stages differed in duration, environmental conditions, sampling design, and analytical endpoints.

In the analyzed within-plot subset, root-associated concentrations exceeded aboveground concentrations for Cu, Zn, Pb, and Mn. Because no uncontaminated mineral-soil control with organ-specific analysis was included, no cultivar-specific background concentrations or normal TF/BCF ranges were established. Residual fine soil particles also cannot be excluded. The results should therefore be interpreted as a descriptive compartment pattern and not as evidence of normal partitioning, metal tolerance, a specific internal accumulation mechanism, or phytostabilization effectiveness.

The study should be interpreted as an exploratory multi-soil screening followed by a site-specific, single-plot pilot assessment. The design does not support site-wide or operational conclusions regarding remediation efficiency. Future studies should include replicated field plots, bioavailable metal fractions, standardized drying procedures, organ-specific laboratory analyses, and longer-term assessment of soil-metal mobility and vegetation persistence.

## Figures and Tables

**Table 1:** Detailed final biometric characteristics of the seven plants selected for destructive assessment from the CA5 pilot plot.

Parameter ^1^	Mean ± SD	Median	Min-Max
Total plant length (cm)	53.29 ± 10.01	55.0	40.0–66.0
Root length (cm)	18.50 ± 4.12	19.5	10.0–22.5
Stem + leaf length (cm) ^2^	34.79 ± 7.55	33.5	23.0–46.5
Mean leaf length (cm)	23.50 ± 6.13	24.0	13.5–33.0
Rosette diameter (cm)	43.86 ± 10.35	46.0	23.0–54.0
Oven-dry biomass (g)	226.83 ± 169.34	171.60	12.18–549.45

^1^ Values describe the seven spatially stratified, purposively selected plants analyzed from the single CA5 pilot plot. The subset was non-random, and the summary statistics are descriptive of the analyzed plants only. The seven plants are within-plot observations and not independent field replicates. ^2^ The stem + leaf measurements in this table refer only to the seven plants selected for destructive assessment and should not be confused with the longitudinal height summaries reported for all 12 surviving plants.

**Table 2:** Baseline edaphic descriptors of the soil variants used in the laboratory screening and field assessment [34].

Soil Variant	Indicative Texture/Material	Soil Structural Class [21] ^2^	Initial pH	Initial EC (mS cm^−1^)
HI	Loamy	Well structured	7.56	0.09
NI	Loamy	Very poorly structured	6.17	0.03
BA1	Loamy	Poorly structured	5.27	0.14
BA2	Loamy	Very well structured	5.59	0.06
BA3	Loamy	Very well structured	6.89	0.79
BO	Loamy	Poorly structured	7.33	0.11
CA1	Loamy	Very well structured	7.09	0.19
CA2	Loamy	Well structured	7.43	0.1
CA3	Loamy	Very well structured	7.33	0.06
CA4	Loamy	Partially structured	7.98	0.08
CA5	Sandy clay	Very well structured	7.31	0.07
BT1	Loamy	Well structured	7.38	0.06
BT2	Loamy	Well structured	6.81	0.07
BT3	Sandy clay	Very poorly structured	5.34	0.07
REF ^1,3^	Commercial peat-based horticultural substrate with perlite, limestone, fertilizer, and root-growth activator	Not applicable	Not measured; manufacturer-declared pH 5.0–6.5 in CaCl_2_	Not determined

^1^ REF was used only as a growth reference commercial substrate. It was not an uncontaminated mineral-soil control and was excluded from soil-property correlation analyses and derived geochemical indicators. ^2^ Soil structural classes were determined using the Sekera method [21]. ^3^ The product label also declared salinity below 3.0 g L^−1^. Manufacturer-declared specifications were not treated as measurements obtained using the study’s soil analytical protocol.

## Data Availability

The data supporting the reported results are provided in Supplementary Tables S1–S7, including baseline pseudo-total soil concentrations, soil- and pot-level plant-performance summaries, individual field measurements, and the concentration data used to calculate BAC, TF, and BCF.

## Supplementary Materials

The following supporting information can be downloaded at: https://www.mdpi.com/article/10.3390/plants15162482/s1, Table S1: Pseudo-total metal concentrations of the mining-affected soils used in the laboratory screening; Table S2: Laboratory establishment, final biometric characteristics, and pooled seven-day air-dried mass of *Brassica oleracea* var. *acephala* ‘Pigeon’ across soil variants; Table S2a: Pot-level establishment at 30 days after sowing and final pot-mean total plant length of *Brassica oleracea* var. *acephala* ‘Pigeon’ across soil variants; Table S3: Whole-plant metal concentrations in pooled seven-day air-dried *Brassica oleracea* var. *acephala* ‘Pigeon’ material from the laboratory screening; Table S4: Descriptive bioaccumulation coefficients (BAC) calculated from whole-plant concentrations and baseline pseudo-total soil concentrations in the laboratory screening; Table S5: Individual final biometric characteristics of the seven plants used for destructive assessment in the single CA5 pilot plot; Table S6: Root-associated and aboveground metal concentrations in the seven field-grown plants selected for chemical assessment in the CA5 pilot plot; Table S7: Translocation factors (TF) and bioconcentration factors (BCF) for the seven field-grown plants from the CA5 pilot plot.

## Abbreviations

The following abbreviations are used in this manuscript:

BAC	Bioaccumulation Coefficient
BCF	Bioconcentration factor
EC	Electrical conductivity
NC	Not calculated
ND	Not detected/below the reporting limit
SD	Standard Deviation

## Appendix A

**Table A1:** Summary of BAC values obtained during the laboratory experiment across mining-affected soil variants.

Metal	BAC Range ^1^	Soil Variants with BAC > 1
Ni	0.318–2.019 (n = 5)	HI, BT2
Cr	1.067–20.860 (n = 7)	HI, BA2, CA1, CA3, CA5, BT1, BT2
Co	0.096	None ^2^
Cu	0.097–2.821 (n = 13)	BA3, CA2
Zn	0.149–2.037 (n = 13)	NI, BA1, BA2
Cd	0.032–0.139 (n = 2)	None
Pb	0.016–0.893 (n = 13)	None
Mn	0.067–0.709 (n = 13)	None

^1^ Ranges were calculated only from soil–metal combinations for which all values required for calculation were quantifiable. Values in parentheses (n) indicate the number of calculated values included in the respective range. Soil concentrations were determined after aqua regia digestion and are therefore interpreted as pseudo-total concentrations. The arithmetic value 1 was not established as a cultivar-specific normal or diagnostic threshold. ^2^ “None” indicates that no calculated BAC value exceeded 1.

## Appendix B. Field Metal Partitioning

**Table A2:** Root-associated and aboveground metal concentrations and descriptive TF and BCF ratios derived in field-grown *Brassica oleracea* var. *acephala* ‘Pigeon’ plants from CA5 soil.

Metal	Root Concentration Range ^1^ (mg kg^−1^)	Aboveground Concentration Range (mg kg^−1^)	TF Range	BCF Range	Interpretation/Statistical Note
Ni	5.333–40.467 (n = 2)	ND (n = 0)	NC (n = 0)	NC (n = 0)	Limited interpretation; aboveground concentrations were not quantifiable.
Cr	0.830–98.267 (n = 7)	0.200–1.933 (n = 2)	0.013–0.020 (n = 2)	0.033–3.869 (n = 7)	Limited interpretation; aboveground concentrations were quantifiable in two plants only.
Co	ND (n = 0)	ND (n = 0)	NC (n = 0)	NC (n = 0)	Not detected in analyzed plant material.
Cu	64.530–229.600 (n = 7)	5.800–18.300 (n = 7)	0.039–0.110 (n = 7)	0.091–0.323 (n = 7)	Root-associated > aboveground; exact paired Wilcoxon test, *p* = 0.0156.
Zn	37.567–731.000 (n = 7)	14.103–306.700 (n = 7)	0.226–0.883 (n = 7)	0.024–0.461 (n = 7)	Root-associated > aboveground; exact paired Wilcoxon test, *p* = 0.0156.
Cd	0.700–1.633 (n = 2)	0.367–0.900 (n = 2)	0.524–0.551 (n = 2)	0.228–0.533 (n = 2)	Limited interpretation; quantifiable in two plants only.
Pb	75.330–445.333 (n = 7)	3.000–25.233 (n = 7)	0.022–0.073 (n = 7)	0.063–0.374 (n = 7)	Root-associated > aboveground; exact paired Wilcoxon test, *p* = 0.0156.
Mn	240.670–1293.333 (n = 7)	36.267–139.500 (n = 7)	0.050–0.259 (n = 7)	0.105–0.566 (n = 7)	Root-associated > aboveground; exact paired Wilcoxon test, *p* = 0.0156.

^1^ Concentration ranges are based on quantifiable values only. Values in parentheses (n) indicate the number of calculated values included in the respective range.
