# Supplementary material for: From Laboratory Screening to a Site-Specific Pilot Field Assessment: Performance of Brassica oleracea var. acephala ‘Pigeon’ on Mining-Impacted Soils
Source: Plants (Basel). 2026 Aug 16;15(16):2482. doi: 10.3390/plants15162482 (PMC13516259; doi:10.3390/plants15162482)
Supplement: Supplementary file 1 [file plants-15-02482-s001.zip › plants-4440649-supplementary.pdf]

Table S1. Pseudo-total metal concentrations of the mining-affected soils used in the laboratory screening.

Soil	Ni [mg kg^{-1}]	Cr [mg kg^{-1}]	Co [mg kg^{-1}]	Cu [mg kg^{-1}]	Zn [mg kg^{-1}]	Cd [mg kg^{-1}]	Pb [mg kg^{-1}]	Mn [mg kg^{-1}]
**HI**	9.90	18.20	ND	295.93	1166.00	10.30	660.00	2160.33
**NI**	ND	6.93	4.83	158.73	442.00	ND	306.90	642.33
**BA1**	ND	ND	2.33	21.57	88.17	ND	31.30	460.67
**BA2**	ND	3.40	ND	38.17	144.47	ND	161.87	1375.33
**BA3**	ND	ND	ND	2.97	238.57	ND	4.63	484.67
**BO**	ND	4.73	1.07	99.67	384.67	ND	241.47	560.33
**CA1**	21.77	14.50	ND	845.67	2300.67	52.83	2337.67	3740.00
**CA2**	ND	ND	5.10	19.60	1127.67	5.80	85.70	340.00
**CA3**	28.57	21.93	ND	93.83	374.33	ND	217.77	1408.00
**CA4**	2.07	ND	7.67	18.97	137.40	ND	22.07	592.00
**CA5**	ND	25.40	10.80	710.00	1584.67	3.07	1192.00	2284.33
**BT1**	4.71	18.67	4.10	59.80	299.40	ND	98.87	547.33
**BT2**	6.36	4.30	7.00	196.10	420.33	ND	365.00	747.67
**BT3**	3.07	22.53	9.70	783.33	1135.00	1.47	860.67	566.33

Notes: Metal concentrations were determined after aqua regia digestion and are interpreted as pseudo-total concentrations. ND denotes a result below the applicable reporting limit and was not interpreted as zero. REF is described in the main manuscript but is not included here because comparable measured soil-metal concentrations were unavailable.

Table S2. Laboratory establishment, final biometric characteristics, and pooled seven-day air-dried mass of *Brassica oleracea* var. *acephala* 'Pigeon' across soil variants.

Soil variant	Germinated seeds [x/6]	Final germination [%]	Plants measured at harvest [n]	Root length mean [cm]	Root length SD [cm]	Stem length mean [cm]	Stem length SD [cm]	Leaf length mean [cm]	Leaf length SD [cm]	Total plant length mean [cm]	Total plant length SD [cm]	Pooled seven-day air-dried mass [g]
**HI**	4/6	66.7	3	10.77	1.56	10.87	1.70	3.07	0.47	24.70	1.11	5.10
**NI**	5/6	83.3	5	12.14	5.21	5.04	1.28	2.92	1.87	20.10	5.35	2.30
**BA1**	5/6	83.3	4	8.08	4.89	6.70	1.21	3.98	1.69	18.75	5.74	3.80
**BA2**	5/6	83.3	6	11.25	4.02	11.33	3.17	3.42	0.58	26.00	6.87	5.40
**BA3**	2/6	33.3	2	18.75	1.06	22.40	4.81	8.35	3.75	49.50	2.12	7.80
**BO**	5/6	83.3	4	3.55	1.73	4.95	4.26	4.00	2.10	12.50	7.84	1.70
**CA1**	5/6	83.3	5	14.72	7.84	9.68	4.97	9.88	4.72	34.28	15.85	5.10
**CA2**	5/6	83.3	6	11.97	2.09	7.68	2.41	6.38	2.55	26.03	4.66	4.10
**CA3**	4/6	66.7	4	12.23	5.45	7.43	2.53	9.20	1.57	28.85	8.76	8.20
**CA4**	6/6	100.0	6	7.00	1.60	3.40	1.20	4.38	1.81	14.78	3.10	1.40
**CA5**	4/6	66.7	4	15.18	7.05	10.73	3.14	8.00	1.64	33.90	8.75	5.80
**BT1**	6/6	100.0	6	8.39	1.98	10.05	2.85	5.05	1.02	23.49	4.43	5.10
**BT2**	6/6	100.0	6	14.92	5.30	11.28	2.36	6.03	0.96	32.23	5.89	9.10
**BT3**	5/6	83.3	3	3.30	0.66	0.63	0.12	2.53	0.76	6.47	0.35	0.90
**REF**	6/6	100.0	6	12.58	4.41	18.02	3.84	4.53	0.67	35.13	6.88	11.90

Notes: Six seeds were sown per soil variant (two seeds in each of three pots). Mass values are pooled seven-day air-dried measurements; constant dry mass was not verified.

Table S2a. Pot-level establishment at 30 days after sowing and final pot-mean total plant length of *Brassica oleracea* var. *acephala* 'Pigeon' across soil variants.

Soil variant	Live seedling at 30 days after sowing			Mean total length [cm]		
	P1	P2	P3	P1	P2	P3
**HI**	2	2	0	24.10	25.90	NA
**NI**	2	1	2	23.40	12.80	20.45
**BA1**	2	2	1	24.00	14.00	23.00
**BA2**	2	2	1	29.00	19.00	30.00
**BA3**	1	0	1	51.00	NA	48.00
**BO**	2	1	2	11.20	20.00	7.60
**CA1**	2	2	1	42.55	24.65	37.00
**CA2**	2	2	1	30.25	26.95	20.90
**CA3**	2	1	1	23.10	38.20	31.00
**CA4**	2	2	2	16.95	16.00	11.40
**CA5**	2	1	1	29.70	41.60	34.60
**BT1**	2	2	2	26.95	22.45	23.50
**BT2**	2	2	2	31.60	35.65	29.45
**BT3**	1	2	2	NA	6.65	6.10
**REF**	2	2	2	38.00	34.00	33.40

Notes: P1–P3 denote the three pots prepared for each soil variant. Pot-level mean total length was calculated across harvested plants within the pot. NA denotes that no plant length was available for that pot; NA was not converted to zero.

Table S3. Whole-plant metal concentrations in pooled seven-day air-dried *Brassica oleracea* var. *acephala* 'Pigeon' material from the laboratory screening.

Soil variant	Ni [mg kg^{-1}]	Cr [mg kg^{-1}]	Co [mg kg^{-1}]	Cu [mg kg^{-1}]	Zn [mg kg^{-1}]	Cd [mg kg^{-1}]	Pb [mg kg^{-1}]	Mn [mg kg^{-1}]
**HI**	15.43	40.37	ND	32.60	173.93	0.33	53.87	246.53
**NI**	ND	ND	ND	39.19	900.29	ND	73.11	91.18
**BA1**	ND	26.90	ND	7.60	88.73	ND	5.50	81.30
**BA2**	0.81	34.23	ND	12.70	290.80	ND	16.80	158.60
**BA3**	ND	4.57	ND	8.37	148.00	ND	0.67	59.67
**BO**	ND	ND	ND	15.33	127.70	ND	23.22	95.61
**CA1**	6.93	15.47	ND	87.80	743.33	7.37	37.73	959.00
**CA2**	ND	9.37	ND	28.27	391.67	ND	76.50	241.17
**CA3**	18.43	40.67	ND	34.37	176.23	ND	49.63	415.33
**CA4**	ND	ND	ND	7.13	25.42	ND	5.58	39.44
**CA5**	4.33	60.07	ND	68.67	358.67	ND	135.10	443.67
**BT1**	3.88	47.47	ND	16.40	82.00	ND	20.63	105.63
**BT2**	12.84	89.70	0.67	123.53	231.00	ND	145.40	360.00
**BT3**	NA	NA	NA	NA	NA	NA	NA	NA

Notes: Roots, stems, and leaves were combined because organ-specific laboratory biomass was insufficient for the target analytical aliquot in several variants. Concentrations are reported on the seven-day air-dried mass basis used for digestion. ND denotes a result below the applicable sample-specific reporting limit and was not interpreted as zero. NA denotes not analyzed because insufficient material was available for the laboratory whole-plant determination.

Table S4. Descriptive bioaccumulation coefficients (BAC) calculated from whole-plant concentrations and baseline pseudo-total soil concentrations in the laboratory screening.

Soil variant	Ni	Cr	Co	Cu	Zn	Cd	Pb	Mn
**HI**	1.559	2.218	NC	0.110	0.149	0.032	0.082	0.114
**NI**	NC	NC	NC	0.247	2.037	NC	0.238	0.142
**BA1**	NC	NC	NC	0.352	1.006	NC	0.176	0.176
**BA2**	NC	10.068	NC	0.333	2.013	NC	0.104	0.115
**BA3**	NC	NC	NC	2.821	0.620	NC	0.145	0.123
**BO**	NC	NC	NC	0.154	0.332	NC	0.096	0.171
**CA1**	0.318	1.067	NC	0.104	0.323	0.139	0.016	0.256
**CA2**	NC	NC	NC	1.442	0.347	NC	0.893	0.709
**CA3**	0.645	1.855	NC	0.366	0.471	NC	0.228	0.295
**CA4**	NC	NC	NC	0.376	0.185	NC	0.253	0.067
**CA5**	NC	2.365	NC	0.097	0.226	NC	0.113	0.194
**BT1**	0.824	2.543	NC	0.274	0.274	NC	0.209	0.193
**BT2**	2.019	20.860	0.096	0.630	0.550	NC	0.398	0.481
**BT3**	NC	NC	NC	NC	NC	NC	NC	NC

Notes: NC denotes not calculated because at least one required value was ND or unavailable. BAC is presented as a descriptive concentration ratio and should not be interpreted as direct evidence of metal removal, phytoavailability, or phytoextraction efficiency. Indices were calculated from unrounded concentration values.

Table S5. Individual final biometric characteristics of the seven plants used for destructive assessment in the single CA5 pilot plot.

Plant	Total plant length (cm)	Root length (cm)	Stem + leaf length (cm)	Mean leaf length (cm)	Rosette diameter (cm)	Oven-dry biomass (g)
V1	66.00	19.50	46.50	33.00	54.00	549.45
V2	55.00	19.00	36.00	20.00	39.50	154.81
V3	56.00	22.50	33.50	28.00	52.00	315.14
V4	40.00	10.00	30.00	13.50	23.00	12.18
V5	62.00	21.00	41.00	24.00	45.00	150.60
V6	54.00	20.50	33.50	24.00	47.50	234.06
V7	40.00	17.00	23.00	22.00	46.00	171.60

Notes: The plot length was divided into seven equal longitudinal subzones. and one plant was selected from each subzone while spanning the observed size range. These plants are within-plot observations and not independent field replicates; the table characterizes the analyzed subset only.

Table S6. Root-associated and aboveground metal concentrations in the seven field-grown plants selected for chemical assessment in the CA5 pilot plot.

Soil variant	Compartment	Ni [mg kg^{-1}]	Cr [mg kg^{-1}]	Co [mg kg^{-1}]	Cu [mg kg^{-1}]	Zn [mg kg^{-1}]	Cd [mg kg^{-1}]	Pb [mg kg^{-1}]	Mn [mg kg^{-1}]
V1	Aboveground	ND	ND	ND	14.07	231.37	ND	7.83	40.27
V1	Root-associated	ND	2.67	ND	163.10	692.33	ND	223.43	681.67
V2	Aboveground	ND	ND	ND	6.20	306.70	ND	4.60	62.33
V2	Root-associated	ND	0.83	ND	64.53	347.33	ND	75.33	240.67
V3	Aboveground	ND	1.93	ND	18.30	20.34	0.90	25.23	139.50
V3	Root-associated	40.47	98.27	ND	229.60	72.73	1.63	445.33	1293.33
V4	Aboveground	ND	0.20	ND	5.80	14.10	0.37	3.00	36.27
V4	Root-associated	5.33	14.87	ND	88.00	37.57	0.70	106.93	480.67
V5	Aboveground	ND	ND	ND	13.47	242.27	ND	10.57	68.47
V5	Root-associated	ND	2.77	ND	122.27	484.67	ND	145.60	385.00
V6	Aboveground	ND	ND	ND	6.77	166.33	ND	9.07	61.17
V6	Root-associated	ND	2.13	ND	128.07	522.33	ND	231.87	622.33
V7	Aboveground	ND	ND	ND	6.07	164.87	ND	4.77	42.07
V7	Root-associated	ND	6.53	ND	155.60	731.00	ND	216.73	833.67

Notes: Concentrations are reported on an oven-dry mass basis. ND denotes a result below the applicable sample-specific reporting limit and was not interpreted as zero. The term root-associated is used because the washing procedure cannot exclude an unknown contribution from fine soil particles or metals adsorbed to root surfaces. The seven plants are paired within-plot observations from one unreplicated pilot plot.

Table S7. Translocation factors (TF) and bioconcentration factors (BCF) for the seven field-grown plants from the CA5 pilot plot.

Plant	Metal	TF	BCF
V1	Ni	NC	NC
V1	Cr	NC	0.105
V1	Co	NC	NC
V1	Cu	0.086	0.230
V1	Zn	0.334	0.437
V1	Cd	NC	NC
V1	Pb	0.035	0.187
V1	Mn	0.059	0.298
V2	Ni	NC	NC
V2	Cr	NC	0.033
V2	Co	NC	NC
V2	Cu	0.096	0.091
V2	Zn	0.883	0.219
V2	Cd	NC	NC
V2	Pb	0.061	0.063
V2	Mn	0.259	0.105
V3	Ni	NC	NC
V3	Cr	0.020	3.869
V3	Co	NC	NC
V3	Cu	0.080	0.323
V3	Zn	0.280	0.046
V3	Cd	0.551	0.533
V3	Pb	0.057	0.374
V3	Mn	0.108	0.566
V4	Ni	NC	NC
V4	Cr	0.013	0.585
V4	Co	NC	NC
V4	Cu	0.066	0.124
V4	Zn	0.375	0.024
V4	Cd	0.524	0.228

V4	Pb	0.028	0.090
V4	Mn	0.075	0.210
V5	Ni	NC	NC
V5	Cr	NC	0.109
V5	Co	NC	NC
V5	Cu	0.110	0.172
V5	Zn	0.500	0.306
V5	Cd	NC	NC
V5	Pb	0.073	0.122
V5	Mn	0.178	0.169
V6	Ni	NC	NC
V6	Cr	NC	0.084
V6	Co	NC	NC
V6	Cu	0.053	0.180
V6	Zn	0.318	0.330
V6	Cd	NC	NC
V6	Pb	0.039	0.195
V6	Mn	0.098	0.272
V7	Ni	NC	NC
V7	Cr	NC	0.257
V7	Co	NC	NC
V7	Cu	0.039	0.219
V7	Zn	0.226	0.461
V7	Cd	NC	NC
V7	Pb	0.022	0.182
V7	Mn	0.050	0.365

Notes: NC denotes not calculated because at least one required concentration was ND or unavailable. Indices are descriptive within-plot values and are not independent site-level replicates. BCF uses the baseline pseudo-total concentration of the CA5 soil and therefore does not represent a ratio to the phytoavailable soil fraction. Indices were calculated from unrounded concentration values.
